# Assessment of the Telomere Length and Its Effect on the Symptomatology of Parkinson’s Disease

**DOI:** 10.3390/antiox10010137

**Published:** 2021-01-19

**Authors:** Tina Levstek, Sara Redenšek, Maja Trošt, Vita Dolžan, Katarina Trebušak Podkrajšek

**Affiliations:** 1 Institute of Biochemistry and Molecular Genetics, Faculty of Medicine, University of Ljubljana, Vrazov trg 2, 1000 Ljubljana, Slovenia; tina.levstek@mf.uni-lj.si (T.L.); sara.redensek@mf.uni-lj.si (S.R.); vita.dolzan@mf.uni-lj.si (V.D.); 2 Department of Neurology, University Medical Centre Ljubljana, Zaloška cesta 2, 1000 Ljubljana, Slovenia; maja.trost@kclj.si; 3 Clinical Institute for Special Laboratory Diagnostics, University Children’s Hospital, University Medical Centre Ljubljana, Vrazov trg 1, 1000 Ljubljana, Slovenia

**Keywords:** Parkinson’s disease, telomere length, telomere attrition, dementia, levodopa treatment

## Abstract

Telomeres, which are repetitive sequences that cap the end of the chromosomes, shorten with each cell division. Besides cellular aging, there are several other factors that influence telomere length (TL), in particular, oxidative stress and inflammation, which play an important role in the pathogenesis of neurodegenerative brain diseases including Parkinson’s disease (PD). So far, the majority of studies have not demonstrated a significant difference in TL between PD patients and healthy individuals. However, studies investigating the effect of TL on the symptomatology and disease progression of PD are scarce, and thus, warranted. We analyzed TL of peripheral blood cells in a sample of 204 PD patients without concomitant autoimmune diseases and analyzed its association with several PD related phenotypes. Monochrome multiplex quantitative PCR (mmqPCR) was used to determine relative TL given as a ratio of the amount of DNA between the telomere and albumin as the housekeeping gene. We found a significant difference in the relative TL between PD patients with and without dementia, where shorter TL presented higher risk for dementia (*p* = 0.024). However, the correlation was not significant after adjustment for clinical factors (*p* = 0.509). We found no correlations between TLs and the dose of dopaminergic therapy when the analysis was adjusted for genetic variability in inflammatory or oxidative factors. In addition, TL influenced time to onset of motor complications after levodopa treatment initiation (*p* = 0.0134), but the association did not remain significant after adjustment for age at inclusion and disease duration (*p* = 0.0781). Based on the results of our study we conclude that TL contributes to certain PD-related phenotypes, although it may not have a major role in directing the course of the disease. Nevertheless, this expends currently limited knowledge regarding the association of the telomere attrition and the disease severity or motor complications in Parkinson’s disease.

## 1. Introduction

Telomeres are heterochromatin structures consisting of tandem repeats of the 5′-TTAGG-3′ sequence that cap the end of each chromosome and function to maintain genome stability [1]. The mean telomere length (TL) in humans ranges from 10,000 to 15,000 nucleotide base pairs [2]. Due to the inability of DNA polymerase to fully replicate the end of linear DNA (the “end replication problem”) [3], telomeres shorten at a rate of 50–200 base pairs per each cell division [4], and telomere shortening, is thus, considered a biomarker of cell ageing. In addition to cellular aging, there are several other factors that can influence TL, especially oxidative stress [5] and inflammation [6]. Due to the high guanine content in the telomeric repeats, telomeres are highly susceptible to oxidative stress damage, which is less well repaired in the telomeric region than elsewhere on the chromosome [5], while chronic inflammation promotes higher cell turnover leading to telomere shortening [6]. It is known that the rate of telomere shortening occurs at the onset of various pathological conditions associated with increased oxidative stress and/or inflammation such as diabetes [7], osteoarthritis [8,9], osteoporosis [9], Alzheimer’s disease (AD) [10], atherosclerosis [11] etc.

Parkinson’s disease (PD) is the second most common neurodegenerative disorder after AD. It is a chronic, progressive and incurable movement disorder. Two of the most important histopathological hallmarks of PD are loss of dopaminergic neurons in the substantia nigra pars compacta and presence of cytoplasmic inclusions (Lewy bodies) mainly composed of α-synuclein in surviving neurons [12,13], which finally lead to akinesia, rigidity and rest tremor [14]. Other symptoms often include various non-motor symptoms like, cognitive, psychiatric and autonomic problems [14]. The full etiology of the disease is still not elucidated [15], however it is known that different genetic [16] and environmental factors play a role. They culminate in increased oxidative stress and neuroinflammation, which lead to further neurodegeneration [17]. Persistently activated microglia release proinflammatory cytokines (interleukin 1β (IL1β), tumor necrosis factor alpha (TNFα) and interleukin-6 (IL6)), chemokines, complement proteins, and reactive oxygen and nitrogen species (ROS, and RNS, respectively), which further enhance the neuroinflammation [18,19,20]. Two additional important ROS production sources are the dopamine metabolism itself [21] and complex I deficiency, which contributes to mitochondrial dysfunction [22]. Genes involved in the processes of oxidative stress and neuroinflammation are highly polymorphic. This genetic variability might affect different disease-related phenotypes [23]. Currently available therapies for PD are symptomatic. Levodopa is a gold standard [24], but its long-term use is associated with reduced efficacy and complications such as motor fluctuations (wearing-off and on-off phenomena), dyskinesia and behavioral abnormalities [25].

Increased oxidative stress, which is also present in the PD brain, promotes telomere attrition mostly through single-strand DNA damage during DNA replication [5]. Therefore, one would expect that telomere attrition in PD patients would be accelerated. However, studies on TL in PD patients yielded contradictory results (reviewed in [26]) and the meta-analysis by Forero et al. [27] showed no significant difference in TLs between PD patients and healthy individuals. TL was mainly investigated in blood cells, while only a few studies investigated TL in brain tissue [26]. 

The aim of this study was to investigate the effect of TL on various clinical characteristics of the PD, that significantly affect patients’ health related quality of life in advanced PD. Since AD, the most common cause of dementia worldwide, was previously associated with a shorter TL [28] we aimed to investigate the correlation between TL and dementia in PD. We hypothesized that patients with shorter TL would be more prone to developing dementia. Furthermore, to our knowledge, no study has investigated the influence of TL on progression of PD. Telomere shortening is associated with neuronal decay and the loss of dopaminergic neurons reflects in the disease severity and in the occurrence of motor complications due to levodopa treatment as well [17,29,30]. Therefore, we aimed to investigate the dose of dopaminergic therapy as an indicator of disease severity and the time to the occurrence of motor complications from levodopa in relation to the TL in PD. We hypothesized that shorter telomeres would predict the disease severity and the development of motor complications.

## 2. Materials and Methods

### 2.1. Design and Subjects

A cohort of PD patients, diagnosed according to the UK Parkinson Disease Society Brain Bank criteria by an experienced movement disorder specialist, was enrolled in the study. The reported study is a sub-study of a project described previously [31] in which 231 PD patients were studied. The recruitment period lasted from October 2016 to April 2018. The inclusion criteria were (1) available clinical data, (2) at least 3 months of levodopa treatment, and (3) ongoing dopaminergic therapy. Patients with atypical and secondary forms of parkinsonism were not included. After exclusion of the patients with autoimmune diseases, study cohort consisted of 204 PD patients (119 men and 85 women). All the demographic data and clinical information, including information on treatment and the occurrence of motor complications after initiation of levodopa is available elsewhere [31]. The criteria for the presence of dementia in individual patient was the need for the anti-dementia medication due to significant cognitive decline.

Peripheral blood samples were previously collected in EDTA tubes. Genomic DNA was isolated from peripheral blood leukocytes. All the patients were genotyped for common functional polymorphisms in antioxidant and inflammatory pathways in a study focusing on the predictive models for time to occurrence of levodopa related motor complications in Parkinson’s disease (*NLRP3* rs35829419, *CARD8* rs2043211, *IL1β* rs16944, *IL1β* rs1143623, *IL6* rs1800795, *CAT* rs1001179, *CAT* rs10836235, *SOD2* rs4880, *NOS1* rs2293054, *NOS1* rs2682826, *TNFα* rs1800629, and *GPX1* rs1050450). The SNP selection and genotyping analysis were carried out as described previously [31]. 

The study protocol was approved by the Slovenian Ethics Committee for Research in Medicine (KME 42/05/16). All subjects gave written informed consent in accordance with the Declaration of Helsinki.

### 2.2. Telomere Length Detection

DNA samples, which had previously been isolated from peripheral blood cells and stored at 4 °C, were used to measure TL [23]. TL detection was performed as initially described by Cawthon [32]. Briefly, isolated DNA was diluted to 15 ng/µL into pure water prior to the start of the experiment. Telomere sequences were amplified using the primers TelG (ACACTAAGGTTTGGGTTTGGGTTTGGGTTTGGGTTAGTGT) and TelC (TGTTAGGTATCCCTATCCCTATCCCTATCCCTATCCCTAACA), while the single-copy gene, encoding albumin, was amplified using the primers AlbF (CGGCGGCGGGCGGCGCGGGCTGGGCGGAAATGCTGCACAGAATCCTTG) and AlbR (GCCCGGCCCGCCGCGCCCGTCCCGCCGGAAAAGCATGGTCGCCTGTT). Reactions were run together in a 384 well optical plate (FrameStar^®^ 384 Well Skirted PCR Plate) on the instrument QuantStudio 7 Flex Real-Time PCR System (Applied Biosystems, Foster City, CA, USA). PCR reactions were set up by aliquoting 8.7 µL of master mix into each reaction well, followed by 1.3 µL of each DNA sample. Each reaction contained: 20 ng DNA, 0.3 µM of each primer and 1× Melt Doctor HMR MasterMix (Thermo Fisher Scientific, Waltham, MA, USA). Samples were measured in triplicates along with a no template control and a positive control, both measured in quadruplicates. A standard curve generated by five concentrations (1.85 ng/µL, 5.56 ng/µL, 16.67 ng/µL, 50 ng/µL and 150 ng/µL) of reference DNA, measured in quadruplicates, was also included in each run to monitor PCR efficiency and generate standard curves used for relative quantitation. We previously optimized the concentration scale of the standard curve to cover the expected range of the samples.

After thermal cycling and raw data collection, QuantStudio™ Real-Time PCR Software was used to generate two standard curves, one for the telomere signal (T) and one for the albumin signal (S). The T value represents the number of copies of telomere repeats, while the S value represents the number of copies of the albumin gene. We then calculated T/S ratio that is proportional to the average TL of the sample. Melting curve was also carefully evaluated to verify specificity of the PCR products. The standard deviation (SD) of the T/S ratio of positive controls between different experiments was calculated (SD = 0.02, data not shown) to ensure that the results from different experiments were comparable.

### 2.3. Statistical Analysis

Median and 25th to 75th percentile range were used to describe central tendency and variability of continuous variables. Frequency and percentage were used to describe categorical variables.

Logistic regression was used to assess the effect of the relative TL on the occurrence of dementia in PD patients as a categorical dependent variable. Odds ratios (ORs), 95% confidence intervals (CIs), and *p*-values were reported. The results of the univariate analysis were adjusted for age at inclusion and disease duration.

Linear regression was used to investigate the association between the relative TL and the current levodopa equivalent dose as a continuous dependent variable. The regression coefficients, 95% CIs, and *p*-values were reported. The results of the univariate analysis were adjusted for age at inclusion, disease duration, and several selected single nucleotide polymorphisms (SNPs) from the oxidative stress and neuroinflammation pathways. 

Cox proportional hazards model was used to estimate the effect of the relative TL on the time to occurrence of motor complications after levodopa treatment initiation. Results were reported as hazard ratios (HR), 95% CIs, and *p*-values. We adjusted the results of the univariate analysis for age at inclusion, and disease duration. We estimated the survival probability in relation to the time from levodopa treatment initiation with Kaplan-Mayer and log-rank test. For the latter patients were grouped according to their relative TL as follows (cutoff = median value of the relative TL): (1) relative TL ≥ 0.94; (2) relative TL < 0.94.

All of the statistical analyses were preformed using R software [33,34,35]. We considered a two-sided *p*-value < 0.05 as statistically significant. 

## 3. Results

### 3.1. Patients Characteristics

A total of 204 patients (119 men and 85 women) were included in the study. General demographic and clinical characteristics are summarized in the Table 1. The patients’ median age at inclusion in the study was 72.5 (65.4–78.0) years, while the median age at diagnosis was 61.7 (54.7–71.1) years. The patients’ characteristics, including the levodopa related motor adverse effects and their frequencies are shown in Table 1 together with the levodopa equivalent dose (LED) at inclusion. The median relative TL in PD patients was 0.94 (0.85–1.10), while the median relative TLs for each clinical characteristic are shown in Table 1.

### 3.2. The Effect of the Relative TL on the Occurrence of Dementia in PD Patients

**Figure 1:**
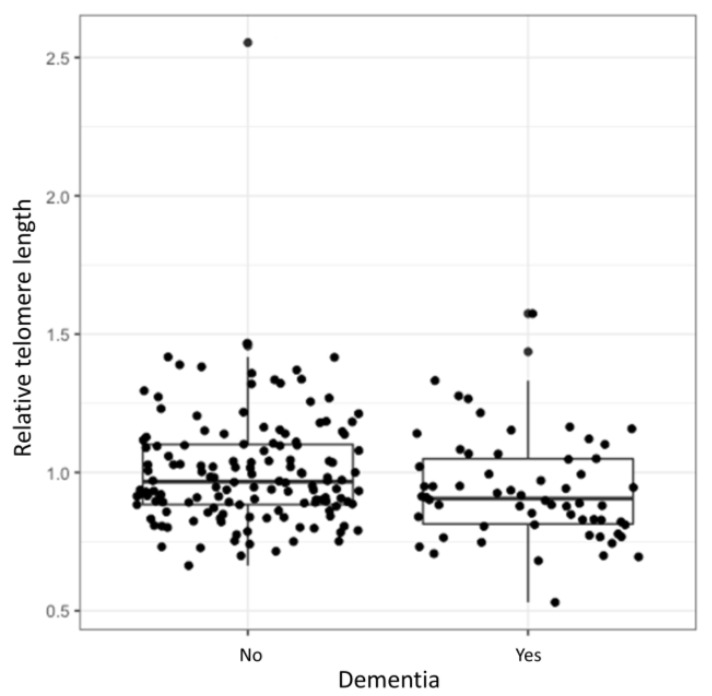
Relative TL in relation to dementia. Median relative TL of a cohort with and without dementia (range between the 1st and 3rd quartile).

Logistic regression showed a statistically significant association between the relative TL and development of dementia (OR = 0.15, 95% CI = 0.03–0.88, *p* = 0.024). Longer TL decreased the odds for the development of dementia. However, after adjustment for age at inclusion and disease duration the correlation was no longer significant (OR = 0.56, 95% CI = 0.097–3.26, *p* = 0.509). Relative TL distributions of patients with and without dementia are presented in the Figure 1. The complete results of the logistic regression analysis are presented in the Supplementary Table S1.

### 3.3. The Effect of the Relative TL on the LED at the Time of Enrolment as an Indicator of the Current Disease State 

**Figure 2:**
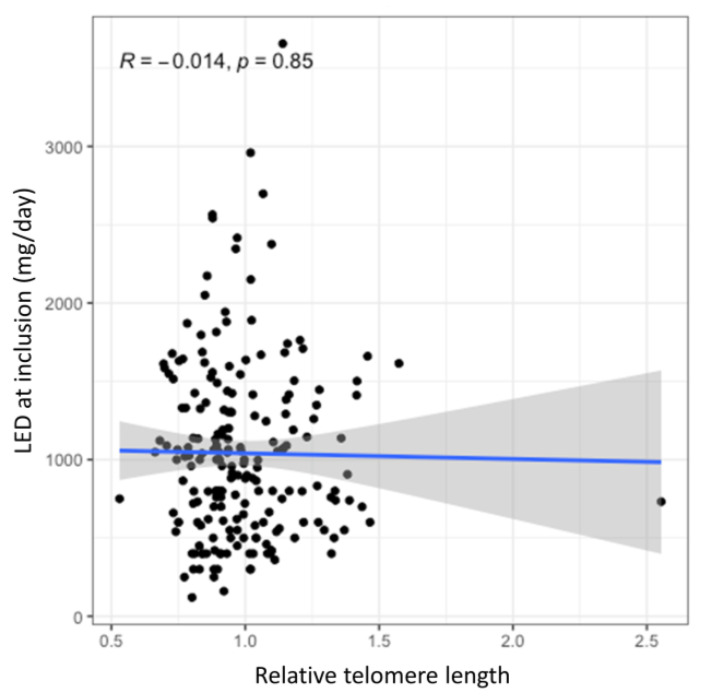
The effect of TL on the current levodopa efficient dose (LED). The blue line presents the regression trendline with the grey area presenting the 95% CI.

No statistically significant association was observed between the relative TL and current dose of dopaminergic therapy neither in the univariate analysis (B = −36.39, 95% CI = −407.7–334.9, *p* = 0.847) nor in the multivariate analysis after adjustment for age at enrolment and disease duration (B = 18.34, 95% CI = −309.11–345.79, *p* = 0.912). Furthermore, this association was also not statistically significant after adjustments for genetic variability in antioxidant and neuroinflammatory pathways (B = 14.48, 95% CI = −317.69–346.65, *p* = 0.932; B = 43.58, 95% CI = −285.74–372.89, *p* = 0.794, respectively). The lack of correlation between the relative TL and the current LED is graphically presented in the Figure 2, whereas complete results of this analysis are presented in the Supplementary Table S2.

### 3.4. The Effect of the Relative TL on the Time to Occurrence of Motor Complications after Levodopa Treatment Initiation

**Figure 3:**
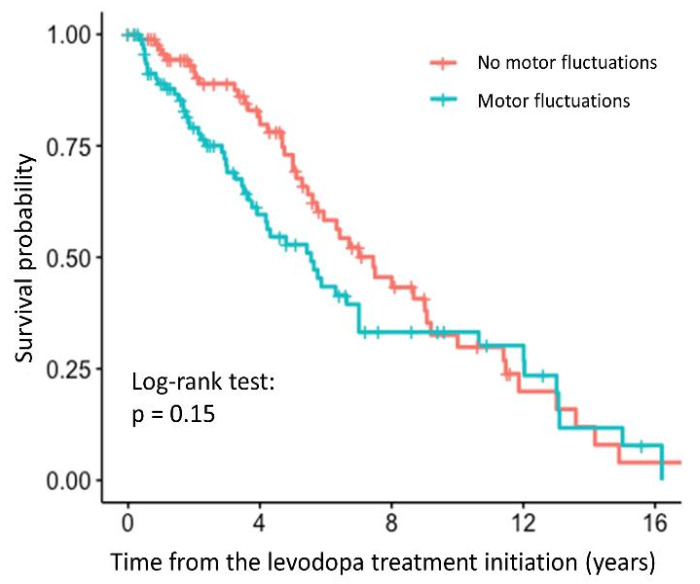
Survival curve for PD patients with and without motor fluctuations after levodopa treatment initiation.

The time to occurrence of motor fluctuations was significantly associated with the relative TL in the univariate analysis (HR = 3.24, 95% CI = 1.28–8.21, *p* = 0.0134). Patients with longer relative TL were more prone to faster development of motor fluctuations. However, the association was no longer significant after adjustment for age at inclusion and disease duration (HR = 2.54, 95% CI = 0.90–7.14, *p* = 0.0781). The survival curve with the result of the log-rank test is shown in the Figure 3, indicating that there is no difference between the patients with and without motor fluctuations, whereas the results of the regression analysis are presented in the Supplementary Table S3.

**Figure 4:**
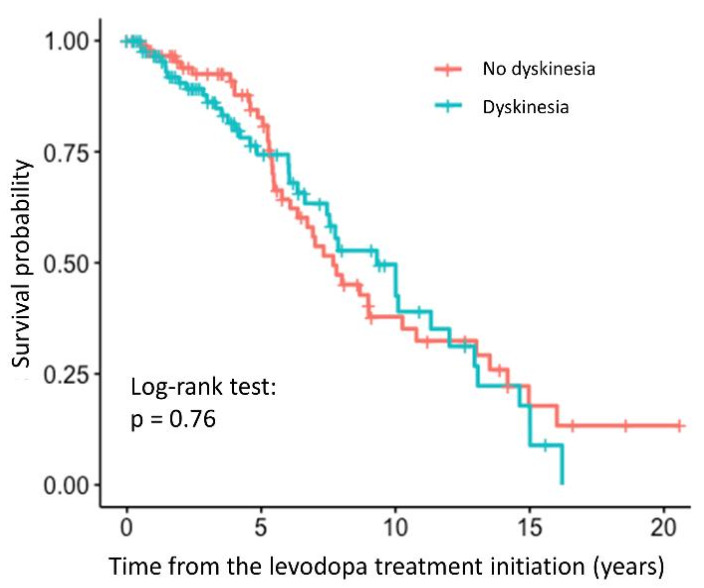
Survival curve for PD patients with and without dyskinesia after levodopa treatment initiation.

We did not observe any statistically significant association between the relative TL and the time to occurrence of dyskinesia (HR = 0.79, 95% CI = 0.23–2.67, *p* = 0.698). The association was not significant even after adjustment for the age at inclusion and disease duration (HR = 0.29, 95% CI = 0.071–1.21, *p* = 0.0904). The survival curve with the result of the log-rank test is shown in the Figure 4, indicating that there is no difference between the patients with and without dyskinesia, whereas complete results of the regression analysis are presented in the Supplementary Table S4.

## 4. Discussion

Our study investigated the associations between TL and some bothersome clinical characteristics of PD; the presence of dementia, the dose requirement of the dopaminergic therapy, and time to occurrence of motor complications after levodopa treatment initiation. Differences in TLs have been suggested as possible risk factors for disorders in which oxidative stress and inflammation are a part of disease pathogenesis. As both contribute significantly to the pathogenesis of PD [17], we were interested in its association with parkinsonian clinical features. So far, studies on the association between TL and the symptomatology of PD or its disease course are scarce; therefore, our study is one of the first to examine this relation.

Cognitive decline is one of the most common non-motor symptoms in PD, its etiology is heterogeneous and has not yet been sufficiently elucidated [36]. Mild cognitive impairment affects about 20–50% of patients with PD [37]. We found a statistically significant association between the relative TL and dementia in PD patients, but after the adjustment for age at enrolment and disease duration, the association was no longer significant. Interestingly, longer TL at diagnosis were previously found to be a risk factor for dementia progression [38]. In contrast, a recent study found shorter telomeres in patients with PD compared to the healthy control group, which also correlated with development of dementia. Namely, shorter telomeres at baseline were found in those PD patients who developed dementia within three years [39]. A causal association between TL and general cognitive function was already shown previously in a meta-study, which studied general population. Shorter telomeres were associated with poorer cognitive performance [40]. In our cohort 30% of patients had developed dementia up until enrolment in the study, already. The loss of statistical significance might have occurred due to a relatively small group of patients with dementia. Additionally, it seems that the age at inclusion and the disease duration are the most important among the evaluated factors in developing dementia. This could contribute to the loss of statistical significance when all of the factors were included in the regression model.

No associations between the relative TL and the current LED were found, neither when evaluated alone nor when antioxidant or inflammatory genetic factors were evaluated concomitantly. The latter was rather unexpected as chronically activated microglia in the PD brain releases pro-inflammatory cytokines and ROS [31] and several SNPs in genes encoding inflammatory factors, such as *IL1*β [41], *TNF* [42], and *IL6* [43] have been associated with PD susceptibility. In addition, several antioxidant and prooxidant enzymes have been associated with PD susceptibility, such as glutathione peroxidase (*GPX1*) [44], catalase (*CAT*) [45] and superoxide dismutase (*SOD*) [46]. The latter three enzymes are the main enzymes that scavenge ROS [23]. *CAT* rs1001179 and *SOD2* rs4880 were previously associated with non-motor adverse effects of dopaminergic treatment [23] as well as with the time to occurrence of dyskinesia [31]. Based on these reported effects of oxidative and inflammatory factors, we analyzed the relative TL in relation to the current LED. To our knowledge no study has investigated this association in PD, yet. However, the patient cohort in the reported study was rather homogenous, in terms of age, which means that the distribution of the relative TL among patients was rather narrow as well. Additionally, previous studies did not show clinically important associations of the oxidative stress and neuroinflammation genetic factors with the PD-related phenotypes. All of this may explain the lack of significant associations in this part of our study.

A statistically significant association between the relative TL and the time to occurrence of motor fluctuations was observed. However, the association did not remain significant after adjustment for age at enrolment and disease duration. There was also no association between the relative TL and the time to dyskinesia development. As the disease progresses more and more dopaminergic neurons die, maybe also due to telomere attrition, which means that other types of neurons, such as serotonergic neurons, overtake the function of dopamine synthesis. These neurons do not have the capacity to autoregulate the dopamine production, which means that dopamine levels are not appropriately regulated [47]. The latter could lead to motor complications. However, in our study, we measured the relative TL in the peripheral blood cells, which might not reflect the relative TL in the central nervous system. This might be the reason, why we did not detect any significant association between the relative TL and the time to development of motor complications. To our knowledge, this is the first study to investigate the potential associations between the relative TL and the time to occurrence of motor complications arising from levodopa treatment.

We are aware that the reported findings have to be considered with caution. Samples were collected from PD patients treated with dopaminergic therapy. The effect of treatment on the TL is not known, which means that it was not possible to take this effect into account. Additionally, TL may show a large intraindividual variability and a single measurement may not be sufficient to undoubtedly estimate TL. Moreover, LED at enrolment in the study was used to estimate the disease progression, which is only a rough estimate. Unfortunately, the unified Parkinson’s Disease rating scale was not used to track the course of the disease in this cohort. Furthermore, we did not use the healthy control samples since those would need to be aged-matched DNA samples isolated at the same time as DNA samples of our cohort with the same isolation method and stored under the same conditions to enable proper comparison of the results as recommended by Lin et al. [48]. Lastly, TL measured in the leukocytes is only a surrogate marker of TL in different tissues. It would be of interest to investigate TL in brain tissue where major changes occur in patients with PD. The accessibility and non-invasiveness of blood sampling compared to brain tissue biopsy and the possibility to implement the peripheral blood TL as a biomarker were the reasons to choose the measurement of TL in peripheral blood leukocytes. An additional aspect of research might be the evaluation of the pre-motor symptoms in relation to the telomere attrition. It would also be of interest to address the relation of autoimmune diseases to PD, its symptomatology, and telomere attrition.

To the best of our knowledge, this is the first study that investigated the effect of the TL on different PD-related clinical features, as previous studies have mostly focused on assessing the risk for the disease development in relation to TL. Besides, this is also the first study to assess TL in a way that, in the case of significant and clinically relevant results, it could later be used as a biomarker of disease progression or a predictor of adverse events’ occurrence, which would open a window for personalized care.

## 5. Conclusions

This study demonstrates the importance of understanding TL dynamics in the pathology of PD. We were able to detect a correlation between TL and dementia in PD patients, but the correlation was not significant after adjustment for clinical factors. Our hypothesis was therefore not completely confirmed. We speculate that age at inclusion and disease duration are stronger determinants of the dementia occurrence than relative TL solely. Contrary to expectations, no correlation was found between TL in combination with the antioxidant and inflammatory genetic factors and the dose of the dopaminergic therapy, although oxidative stress and neuroinflammation contribute to the PD pathogenesis. The results indicate a possible association of the relative TL with the time to occurrence of motor fluctuations due to levodopa treatment but not to the occurrence of dyskinesia. Hence, the current study provides an important insight into the vast heterogeneity of symptoms in PD patients. The TL has been recognized as a vital player in certain neurodegenerative diseases, but the evidence proving their role in PD up to date is rather shaky. Further studies with a larger PD cohort are needed to draw firm conclusions about the role of telomere attrition and TL in PD-related clinical features. Only then can TL as a biomarker could help predict the course of the disease and improve the treatment outcome. Nevertheless, TL is probably only one piece of the puzzle that, together with other biomarkers such as genomics, epigenetics and metabolomics biomarkers, could enable the most efficient treatment of patients with PD.

## Figures and Tables

**Table 1:** Patients characteristics.

Patient Characteristic (N = 204)		(% or the Range between the 1st and 3rd Quartile)
Age at enrolment		72.5 (65.4–78.0)
Sex	Men	119 (0.58)
	Women	85 (0.42)
Hypercholesterolemia	No	162 (0.79)
	Yes	42 (0.21)
Hypertension	No	125 (0.61)
	Yes	79 (0.39)
Age at diagnosis		61.7 (54.7–71.1)
Disease duration		7.6 (4.1–13.7)
Dementia	No	142 (0.70)
	Yes	62 (0.30)
Diabetes	No	190 (0.93)
	Yes	14 (0.07)
LED		986.2 (600.0–1351.9)
Motor fluctuations	No	94 (0.46)
	Yes	110 (0.54)
Dyskinesia	No	113 (0.55)
	Yes	91 (0.45)
Relative telomere length *		0.94 (0.85–1.10)
Dementia	No	0.97 (0.88–1.10)
	Yes	0.91 (0.81–1.05)
Motor fluctuations	No	0.93 (0.84–1.08)
	Yes	0.95 (0.86–1.10)
Dyskinesia	No	0.95 (0.88–1.09)
	Yes	0.94 (0.84–1.08)

* The relative TL is given as a ratio in the DNA quantity between the telomere and albumin as the housekeeping gene.

## Data Availability

The data presented in this study are available in supplementary material.

## Supplementary Materials

The following are available online at https://www.mdpi.com/2076-3921/10/1/137/s1, Table S1: The effect of the relative TL on the occurrence of dementia in PD patients, Table S2: The effect of the relative TL on the current LED which reflects the symptomatology of Parkinson’s disease. Table S3: Effect of the relative TL on the time to occurrence of motor fluctuations. Table S4: Effect of the relative TL on the time to occurrence of dyskinesia.

## Abbreviations

TL	telomere length
PD	Parkinson’s disease
ROS	reactive oxygen species
RNS	reactive nitrogen species
LED	levodopa equivalent dose
SNP	single nucleotide polymorphism
