# Supplementary material for: Assessment of the Telomere Length and Its Effect on the Symptomatology of Parkinson’s Disease
Source: Antioxidants (Basel). 2021 Jan 19;10(1):137. doi: 10.3390/antiox10010137 (PMC7835735; doi:10.3390/antiox10010137)
Supplement: Supplementary file 1 [file antioxidants-10-00137-s001.pdf]

Supplementary Material

Table S1: The effect of the relative TL on the occurrence of dementia in PD patients.

	OR	95%CI	p-value
**Telomere length**	0.15	0.03–0.88	**0.024**

Adjustment for age at inclusion and disease duration

	OR	95%CI	p-value
**Telomere length**	0.56	0.097–3.26	0.509
**Age at inclusion**	1.18	1.11–1.25	**<0.001**
**Disease duration**	1.13	1.07–1.19	**<0.001**

Table S2: The effect of the relative TL on the current LED which reflects the symptomatology of Parkinson's disease.

	B	95% CI	p-value
**Telomere length**	-36.39	-407.7–334.9	0.847

Adjustment for age at inclusion and disease duration

	B	95% CI	p-value
**Telomere length**	18.34	-309.11–345.79	0.912
**Age at inclusion**	-9.87	-16.91 –-2.83	**0.00623**
**Disease duration**	38.32	28.44 –48.20	**9.07e-13**

Adjustment for the genetic factors of the oxidative stress pathway

	B	95% CI	p-value
**Telomere length**	14.48	-317.69–346.65	0.932
**Age at inclusion**	-9.96	-17.09 –-2.82	**0.00649**
**Disease duration**	38.48	28.45 –48.51	**1.55e-12**
***GPX1* rs1050450**	-22.29	-133.36–88.78	0.693
***CAT* rs10836235**	-11.82	-157.59–133.96	0.873
***CAT* rs1001179**	-17.13	-141.05–106.79	0.785
***SOD2* rs4880**	26.94	-69.82–123.71	0.584

Adjustment for the genetic factors of the inflammation pathway

	B	95% CI	p-value
**Telomere length**	43.58	-285.74–372.89	0.794
**Age at inclusion**	-9.70	-16.77–-2.61	**0.00760**
**Disease duration**	37.63	27.60–47.66	**4.08e-12**
***IL1B* rs16944**	13.68	-140.2–167.62	0.861
***IL1B* rs1143623**	104.55	-61.12–270.23	0.215
***TNFa* rs1800629**	1.67	-129.13–132.47	0.980
***IL6* rs1800795**	-62.03	-167.78–43.73	0.249

Table S3: Effect of the relative TL on the time to occurrence of motor fluctuations.

	HR	95% CI	p-value
**Telomere length**	3.24	1.28–8.21	**0.0134**

Adjustment for age at inclusion and disease duration

	HR	95% CI	p-value
**Telomere length**	2.54	0.90–7.14	0.0781
**Age at inclusion**	0.95	0.93–0.97	**5.34e-06**
**Disease duration**	0.98	0.94–1.015	0.215

Table S4: Effect of the relative TL on the time to occurrence of dyskinesia.

	HR	95% CI	p-value
**Telomere length**	0.79	0.231–2.67	0.698

Adjustment for age at inclusion and disease duration

	HR	95% CI	p-value
**Telomere length**	0.294	0.071–1.21	0.0904
**Age at inclusion**	0.931	0.91–0.95	**2.27e-09**
**Disease duration**	0.958	0.92–1.0013	0.0571
